# Supplementary material for: Intra-colony channel morphology in Escherichia coli biofilms is governed by nutrient availability and substrate stiffness
Source: Biofilm. 2022 Sep 26;4:100084. doi: 10.1016/j.bioflm.2022.100084 (PMC9568850; doi:10.1016/j.bioflm.2022.100084)
Supplement: Multimedia component 1 [file mmc1.docx]

# Additional information


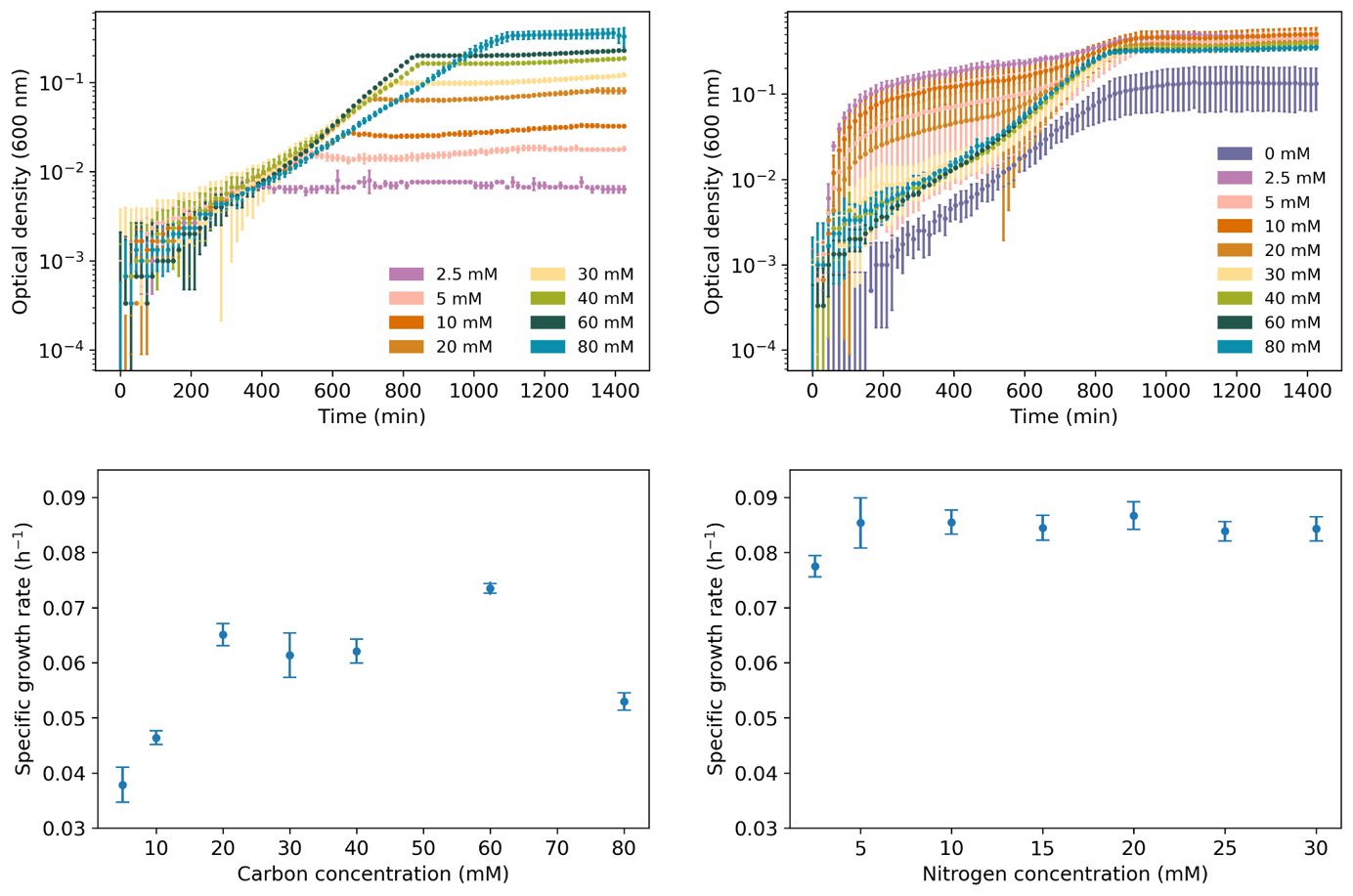


Supplementary Figure 1: Growth curves (top panels) and specific growth rates (bottom panels) of *E. coli* JM105mini-Tn7-*gfp* liquid cultures grown in M9 minimal medium with various carbon (a, c) and nitrogen (b, d) concentrations, obtained by varying the amounts of glucose and ammonium chloride in the medium. Error bars on growth curve plots represent the standard deviation across three biological repeats. The growth curve for the lowest carbon concentration in a (0 mM) is not shown as it consists of a baseline of non-growing cells. The data point at 80 mM carbon (c) is likely due to saturation, especially if we consider that the nominal carbon concentration in M9 medium is 67 mM.


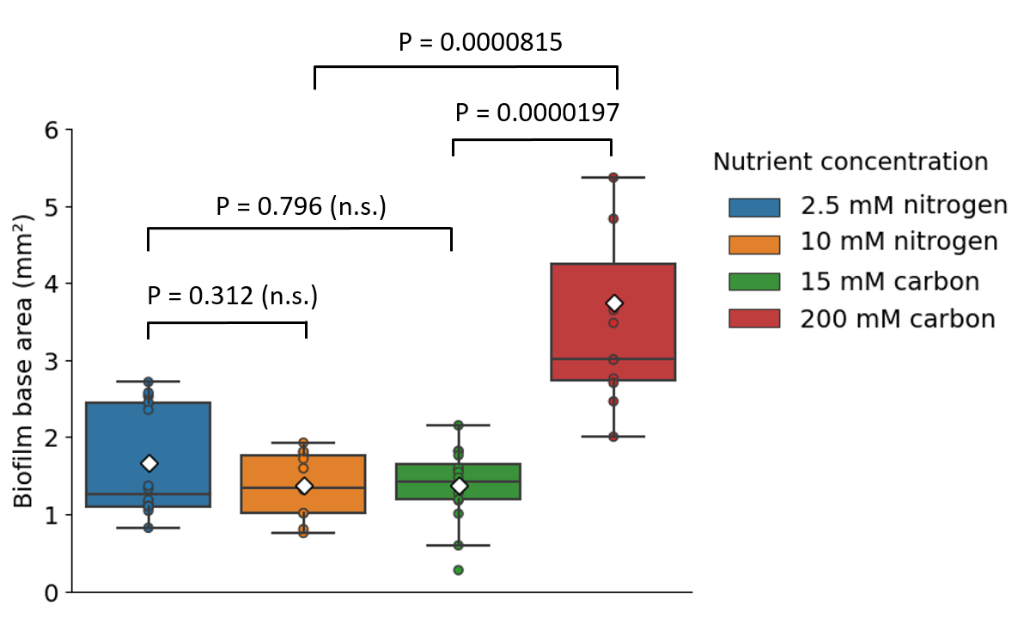


Supplementary Figure 2: Base area of *E. coli* JM105-mini-Tn7-*gfp* biofilms calculated for four nutrient conditions. Average biofilm areas are 1.374 ± 0.118 mm^2^ (glucose-limited biofilms, n = 16), 3.738 ± 0.510 mm^2^ (glucose-rich biofilms, n = 11), 1.668 ± 0.166 mm^2^ (ammonium-limited biofilms, n = 18) and 1.370 ± 0.129 mm^2^ (ammonium-rich biofilms, n = 11). Uncertainties correspond to standard errors of the mean across biological repeats (n ≥ 11 for each condition). Mann-Whitney U rank tests were performed on the data, with relevant p-values shown. Average values are shown as white diamonds, whereas boxes represent the interquantile range (with median values shown as horizontal lines inside each box).


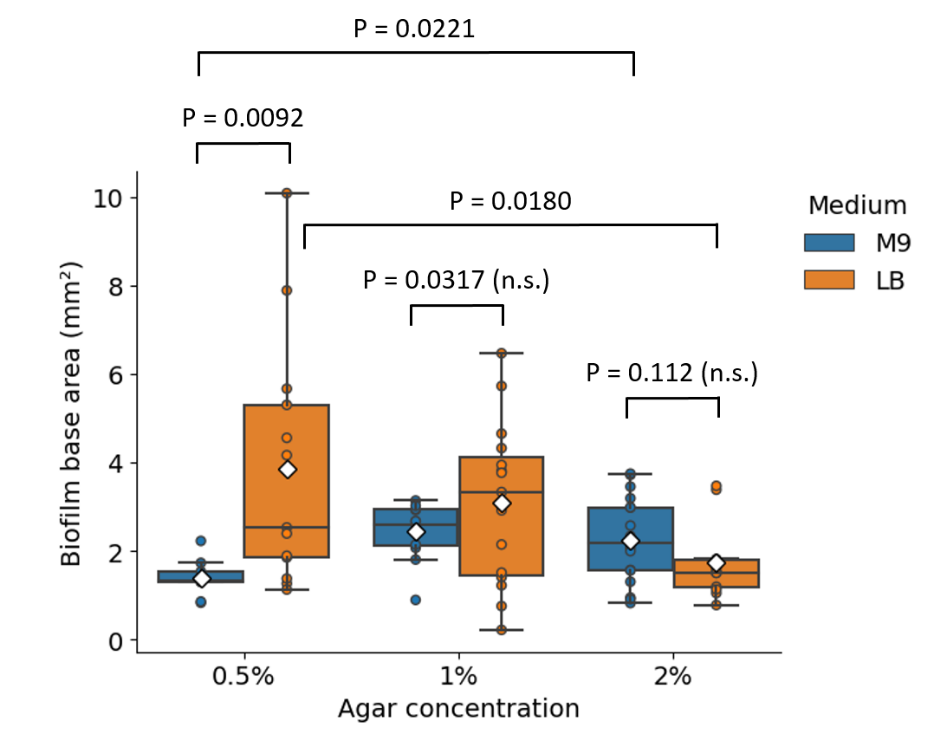


Supplementary Figure 3: *E. coli* JM105-mini-Tn7-*gfp* biofilm base area calculated for three different agar concentrations of the substrate, in both rich (LB) and minimal (M9) medium. Average areas of biofilms grown on rich LB medium are 3.854 ± 0.772 mm^2^ (0.5% agar, n = 13 biofilms), 3.080 ± 0.477 mm^2^ (1% agar, n = 15 biofilms) and 1.733 ± 0.245 mm^2^ (2% agar, n = 12 biofilms). Average areas of biofilms grown on minimal M9 medium are 1.387 ± 0.142 mm^2^ (0.5% agar, n = 9 biofilms), 2.434 ± 0.184 mm^2^ (1% agar, n = 12 biofilms) and 2.253 ± 0.224 (2% agar, n = 18 biofilms). Uncertainties correspond to standard errors on the mean across biological repeats. Mann-Whitney U rank tests were performed on the data, with relevant p-values shown. Average values are shown as white diamonds, whereas boxes represent the interquantile range (with median shown as a horizontal line).


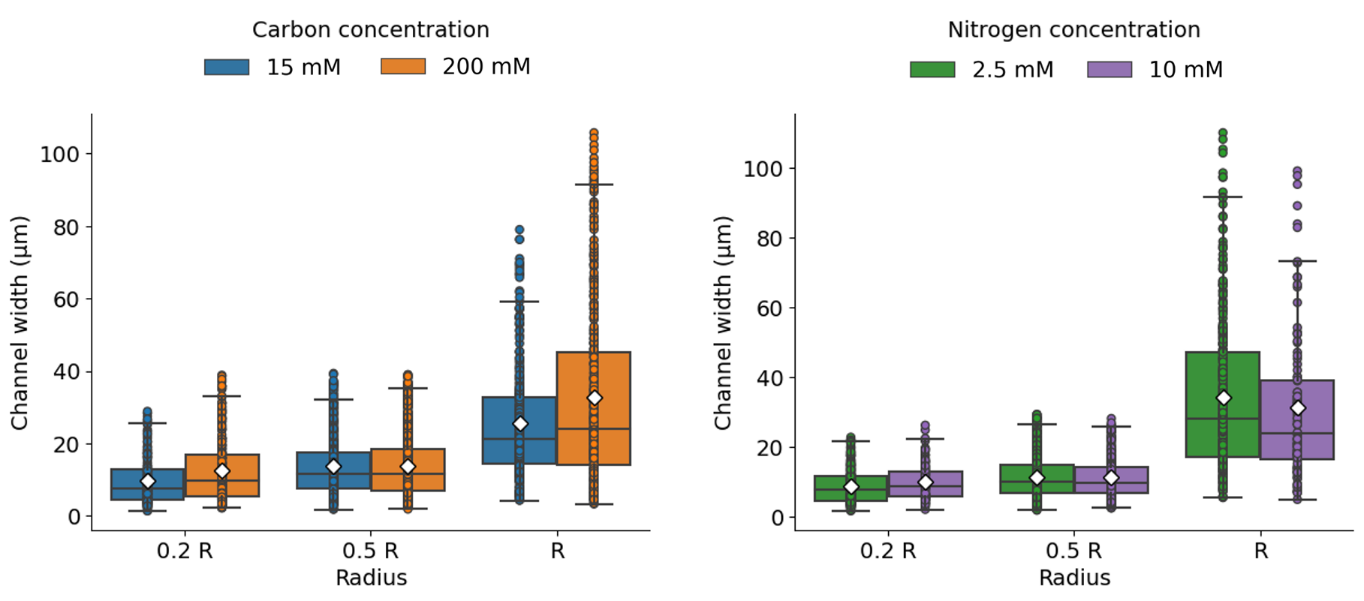


Supplementary Figure 4: Intra-colony channel width calculated at three normalized radial positions (20% radius, 50% radius and full radius, labelled as 0.2 R, 0.5 R and R respectively). Channels were approximately 25% wider on glucose-limited substrates than on ammonium-limited substrates at the mid-radius region of each biofilm, where channel widths measured on average 13.78*µ*m under glucose limitation and 11.27*µ*m under ammonium limitation.


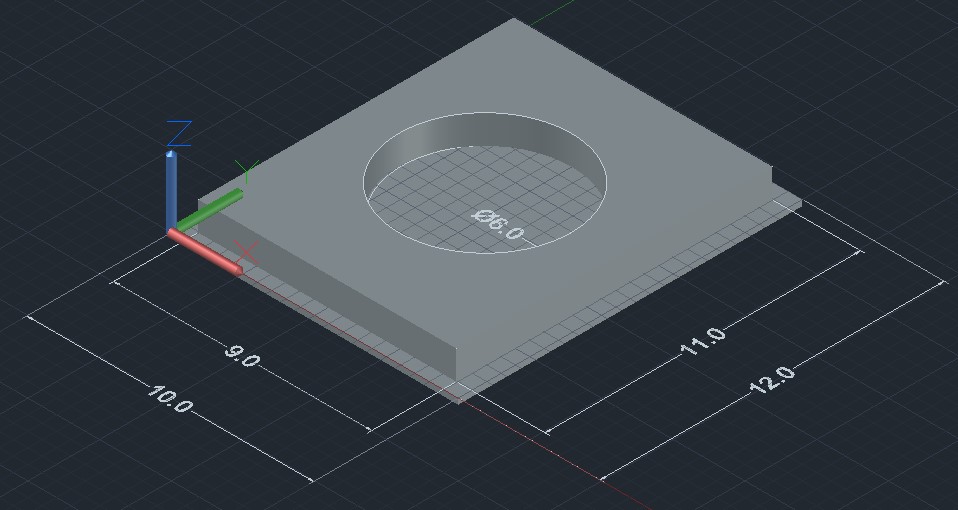


Supplementary Figure 5: 3D-printed imaging chamber used with the Mesolens. Measurements are given in cm.
